# Exploring the Boundaries of Reality: Investigating the Phenomenon of Artificial Intelligence Hallucination in Scientific Writing Through ChatGPT References

**DOI:** 10.7759/cureus.37432

**Published:** 2023-04-11

**Authors:** Sai Anirudh Athaluri, Sandeep Varma Manthena, V S R Krishna Manoj Kesapragada, Vineel Yarlagadda, Tirth Dave, Rama Tulasi Siri Duddumpudi

**Affiliations:** 1 Medicine, Rangaraya Medical College, Kakinada, IND; 2 Internal Medicine, Bukovinian State Medical University, Chernivtsi, UKR

**Keywords:** ai hallucination, gpt-3, natural language processing, artificial intelligence, chatgpt

## Abstract

Background

Chatbots are computer programs that use artificial intelligence (AI) and natural language processing (NLP) to simulate conversations with humans. One such chatbot is ChatGPT, which uses the third-generation generative pre-trained transformer (GPT-3) developed by OpenAI. ChatGPT has been praised for its ability to generate text, but concerns have been raised about its accuracy and precision in generating data, as well as legal issues related to references. This study aims to investigate the frequency of AI hallucination in research proposals entirely drafted by ChatGPT.

Methodology

An analytical design was employed to investigate AI hallucination by ChatGPT. A total of 178 references listed by ChatGPT were verified for inclusion in the study. Statistical analysis was performed by five researchers who entered their data into a Google Form, and the final results were represented using pie charts and tables.

Results

Out of the 178 references analyzed, 69 references did not have a Digital Object Identifier (DOI), and 28 references neither turned up on Google search nor had an existing DOI. Three references were listed from books and not research articles. These observations suggest that ChatGPT’s ability to generate reliable references for research topics may be limited by the availability of DOI and the accessibility of online articles.

Conclusions

The study highlights the potential limitations of ChatGPT’s ability to generate reliable references for research proposals. AI hallucination is a problem that may negatively impact decision-making and may give rise to ethical and legal problems. Improving the training inputs by including diverse, accurate, and contextually relevant data sets along with frequent updates to the training models could potentially help address these issues. However, until these issues are addressed, researchers using ChatGPT should exercise caution in relying solely on the references generated by the AI chatbot.

## Introduction

Chatbots are software programs that simulate conversations with humans using artificial intelligence (AI) and natural language processing (NLP) techniques [1]. One popular example of NLP is the third-generation generative pre-trained transformer (GPT-3) model, which can generate text of any type. OpenAI developed ChatGPT, a chatbot that uses the GPT-3 model, which reached 1 million users within its first week of release in November 2022 [2]. Although ChatGPT is considered a valuable tool for researchers and writers, some researchers have raised concerns about its accuracy and precision, as well as its legal implications and references [3]. AI hallucination is a phenomenon where AI generates a convincing but completely made-up answer. OpenAI’s notes acknowledge that the answers generated by ChatGPT may sound plausible but be nonsensical or incorrect [4]. While several studies have evaluated ChatGPT’s ability to generate research proposals, to our knowledge, none have assessed the validity of the references it generates [5].

This study aims to evaluate the frequency of AI hallucination in research proposals entirely drafted by ChatGPT.

## Materials and methods

The study commenced with the creation of a new chatbox in ChatGPT. A new chatbox was necessary to negate the influence of previous searches or commands on the current results generated by ChatGPT as the results vary according to previous inputs given to the chatbox.

Then the AI Chatbot was instructed to suggest 50 novel medical research topics that can be performed by undergraduate medical students in India. The text input command given to the AI Chatbot was “Suggest 50 novel medical research topics that can be performed by undergraduate medical students in India. The topics must be feasible, interesting, novel ethical and relevant.” ChatGPT suggested 50 such research topics as instructed.

A few examples of the topics that were suggested by ChatGPT include “the impact of technology on doctor-patient relationships,” “the effect of exercise on cognitive function,” and the impact of outdoor green spaces on mental health.”

To set standards for the topics necessary for inclusion in the study, FINER (Feasible, Interesting, Novel, Ethical, and Relevant) criteria were used to refine the pool of 50 topics suggested by ChatGPT (and to consider instructing ChatGPT to suggest more research topics which satisfied the FINER criteria if all the 50 initially suggested topics did not satisfy the FINER criteria as per five independent reviewers).

The FINER criteria is a widely used structural criterion that helps scientists in formulating research questions that are effective and practical. It includes aspects such as feasibility (wherein the research questions should be answerable with the given time, resources, and expertise available to the researcher), interest (wherein interesting research questions should be considered, which are aligned with practical and broader interests), novelty (wherein research questions address gaps in knowledge and add to existing knowledge rather than attempting to re-invent the wheel), ethical considerations (which require the research to comply with the necessary safety and confidentiality measures and protocols, especially when the research involves human beings), and relevance. By following these criteria, researchers can ensure that their questions are well-formulated and that their research has a significant impact.

Five researchers manually assessed whether the suggested topics adhered to the FINER criteria or not, and all agreed that the 50 topics adhered to the FINER criteria.

Then ChatGPT was instructed to write an elaborate research protocol on each of the 50 topics with a proper introduction, objectives, methodology, implications, and references and to provide a Digital Object Identifier (DOI) for all references.

For each of the 50 topics, a research protocol was generated by ChatGPT with a proper introduction, objectives, methodology, implications, and references, and a DOI was provided for some of the references generated. In cases wherein the DOI was not provided by ChatGPT as instructed in the initial command, an additional command was given to ChatGPT to provide DOIs for the generated references in each case, upon which ChatGPT provided all DOIs. All 178 references and their DOIs were verified independently by five researchers through an internet search on Scopus, Google, and PubMed search engines. Personalization was turned off to minimize bias during the internet search. Each researcher noted down their findings as to whether the DOI of each reference was functional and truly existed or not, and whether the reference article itself truly existed or not by searching in Scopus, PubMed, and Google search engines. The DOI, reference, and the title of the study as present in the reference were separately searched for on the internet, and the appearance of any part of the article, abstract, or even the full text was considered as an indication of a true, non-AI-hallucinated reference. The results of all five researchers were then compared and there were five discrepancies. The five discrepancies were then discussed and resolved. An inter-rater reliability analysis was also conducted using Fleiss’ kappa for multiple raters and Krippendorff alpha inter-rater reliability tests to gauge the reliability of the results of all five researchers. The final data were then entered into a Google Form and an Excel sheet was generated, which was inserted into SPSS (IBM Corp., Armonk, NY, USA) for statistical analysis. No ethical approval was required as the study did not involve any human or animal subjects.

## Results

The Krippendorff alpha inter-rater reliability test (Table 1) showed strong reliability (alpha value = 0.9589), and the Fleiss’ Kappa (Table 2) also showed strong reliability (kappa = 0.959, p-value <0.001) of the data collected independently by the five researchers. ChatGPT provided 50 research proposals adhering to the FINER criteria. However, in some cases, ChatGPT did not provide complete proposals or references and abruptly stopped in the middle, without completely obeying the initial command. In such cases, additional commands had to be given to ChatGPT to complete the proposal or reference and provide the DOI.

**Table 1:** Krippendorff alpha inter-rater reliability test. LL: lower limit; UL: upper limit; CI: confidence interval Values range from 0 and 1, where 0 is perfect disagreement and 1 is perfect agreement. Values of the Krippendorff alpha test result range from 0 to 1, where 0 is perfect disagreement and 1 is perfect agreement. Krippendorff suggests that it is customary to require an alpha greater than 0.800, but where tentative conclusions are still deemed acceptable (as in communication research for instance), an alpha value greater than 0.667 is the accepted lowest conceivable limit.

	Alpha	LL 95% CI	UL 95% CI	Units	Observers	Pairs
Nominal	0.9589	0.9403	0.9753	178.0000	5.0000	1,780.0000

**Table 2:** Fleiss’ kappa inter-rater reliability test. ^a^: Sample data contains 178 effective subjects and five raters; z: Z-score for the kappa statistic; Sig.: significance Landis and Koch suggest the following interpretations of Kappa values: <0 - less than chance agreement; 0.01-0.20 - slight agreement; 0.21-0.40 - fair agreement; 0.41-0.60 - moderate agreement; 0.61-0.80 - substantial agreement; 0.81-0.99 - almost perfect agreement

Overall agreement^a^						
	Kappa	Asymptotic			Asymptotic 95% confidence interval	
		Standard error	z	Sig.	Lower bound	Upper bound
Overall agreement	0.959	0.024	40.452	<0.001	0.912	1.005

**Figure 1:**
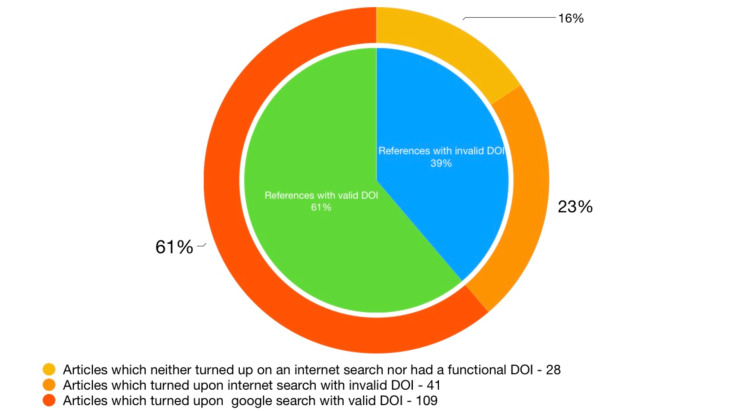
Validity and accessibility of references.

Out of the 178 references cited by ChatGPT, 69 did not have a DOI. Upon extensive internet search, 41 out of these 69 reference articles were found to exist. However, 28 articles neither turned up on Google search nor had an existing DOI. Figure 1 and Table 3 present the obtained results. Additionally, seven references were given from websites of organizations such as the Centers for Disease Control and Prevention (CDC), World Health Organization (WHO), National Institute for Health and Care Excellence (NICE), National Center for Complementary and Integrative Health (NCCIH), and European Association for the Study of the Liver, while three references were listed from books and not research articles. These observations suggest that ChatGPT’s ability to generate reliable references for research topics may be limited by the availability of DOI and the accessibility of online articles.

**Table 3:** Validity and accessibility of references.

Nature of reference	Number of references
References with a valid DOI	109
References with an invalid DOI	69
References that turned up on Google search	150
References that did not turn up on Google search	28

## Discussion

Out of the 69 references for which the DOI did not work at all (that is, the DOI provided by ChatGPT for that reference was non-existent in reality or belonged to a different article), 41 articles that turned up on on an internet search are considered a result of partial AI hallucination. These 41 articles cannot strictly be considered products of total AI hallucination because the articles did exist in reality and only the DOIs provided by ChatGPT in these cases were erroneous.

The 28 articles that neither turned up on an extensive internet search nor had a functional DOI are undoubtedly the product of AI hallucination. Generally, research proposals include journal articles but not miscellaneous websites unless absolutely necessary. ChatGPT cited seven websites and three textbook chapters as references, even when there were scientific journal articles that served the same purpose and provided the exact same information. Though not a blunder, it is more appropriate for scientific journal articles to be included in references rather than websites and chapters from books.

AI hallucination usually occurs due to adversarial examples such as varied input data that confound the AI systems into misclassifying and misinterpreting them resulting in inappropriate and hallucinating output. AI hallucination is a problem because it hampers a user’s trust in the AI system, negatively impacts decision-making, and may give rise to several ethical and legal problems. Improving the training inputs by including diverse, accurate, and contextually relevant data sets along with frequent user feedback and incorporation of human reviewers for evaluation of outputs generated by an AI system are some solutions to this problem of AI hallucination.

In the case of ChatGPT, the expansion of its knowledge base from 2021 to a much more recent time could potentially resolve a lot of AI hallucination incidents occurring through ChatGPT.

The dynamic and continuously evolving nature of AI learning makes it challenging to ensure the credibility of the information generated by AI models. Several factors can contribute to AI hallucination, including differences in the source content and data sets used for training. Various language models, such as GPT-2, Bidirectional Encoder Representations from Transformers (BERT), Robustly Optimized BERT (RoBERTa), XLNet, Text-to-Text Transfer Transformer (T5), and Unified Language Model (UniLM), are trained similarly using comparable data sets, but each model has its strengths and weaknesses. Training of these models is not entirely supervised, and they are not entirely taught what the correct answers are, leading to biases and imprecise decoding from the neural network architecture. Additionally, performance degradation can occur due to changes in data over time, concept drift, software bugs, as well as the aging of the AI model. All of these factors play a pivotal role in the generalization of the results of this study to other AI systems beyond ChatGPT.

The study has several limitations that need to be acknowledged. First, it only focuses on the validity of references generated by ChatGPT and does not assess other potential errors or limitations in the proposals. Second, the sample size of 50 research proposals used in the study is relatively small and may not be representative of the entire range of topics or research areas in which ChatGPT is capable of generating proposals. Moreover, the reliance on Google search to verify the validity of references may not be entirely reliable, as some valid references may not be indexed or are behind paywalls. The results are less reproducible as the AI systems are dynamic and the Google data centers constantly add data to the search engine. Finally, the study also does not incorporate feedback from domain experts or potential users of ChatGPT-generated research proposals.

## Conclusions

AI Hallucination is an area of concern that limits the use of ChatGPT in scientific writing and analysis. The phenomenon may be a formidable burden, but its magnitude can certainly be minimized. Improving the training inputs for AI models by using verified, accurate, and contextually relevant data sets rather than simply a large volume of data, as well as providing continuous user feedback from credible sources on a nominal scale can help reduce drastic data drifts and temporal degradation issues, and, in this case, AI hallucination. However, re-training models and early recognition of deterioration can pose challenges such as catastrophic forgetting and failure to converge, especially in high-risk avenues such as healthcare and scientific literature. Therefore, it is important to conduct more research to identify patterns of inaccurate or missing references beyond the scope of this paper. While ChatGPT and other AI systems can be trusted, they should not be solely relied upon for generating research proposals intended for medical or scientific literature. The use of ChatGPT in scientific writing must be accompanied by acceptance of liability by individual authors for its erroneous results. This study is the first of its kind to analyze the phenomenon of AI hallucination in AI-generated scientific literature and paves the way for further studies to evaluate other aspects of AI hallucination.
